# F9. ALTERATIONS OF NEURONAL METABOLISM IN PATIENT SUBGROUPS AT ULTRA-HIGH RISK FOR PSYCHOSIS ACCORDING TO PACE CRITERIA – A 1H/31P-MR-SPECTROSCOPY STUDY

**DOI:** 10.1093/schbul/sby017.540

**Published:** 2018-04-01

**Authors:** Stefan Smesny, Stephan Schack, Alexander Gussew, Kerstin Langbein, Juergen Reichenbach

**Affiliations:** University Hospital Jena

## Abstract

**Background:**

Glutamatergic dysfunction, deregulated mitochondrial metabolism and alterations of membrane phospholipids have been extensively investigated in schizophrenic illness by using in vivo magnetic resonance spectroscopy (MRS). Findings in the ultra-high risk (UHR) phase of psychotic illness, however, are still rare and inconsistent. Combining both 1H- and 31P-MRS, this study investigates these aspects in the different UHR patient subgroups as defined by PACE (Personal Assessment and Crisis Evaluation) criteria.

**Methods:**

We applied 3 T chemical shift imaging (3D 31P-MRS, 2D 1H-MRS) and hippocampal single-voxel MRS in 69 neuroleptic-naïve UHR patients (age: 26.2 ± 6.2y; males 59.4% 41/69, attenuated symptoms (AS) n=50, BLIPS n=5, genetic risk (GR) n=8, AS+GR n=6; transition rate 17.2%, all transitions in the AS or BLIPS group) and 61 healthy controls (age: 25.2 ± 4.8y; males 54.1% 33/61). 11 metabolite markers were investigated (neuronal/mitochondrial metabolism: glutamate (Glu), N-acetyl-aspartate (NAA), phosphocreatine (PCr), and adenosine triphosphate (ATP); phospholipid synthesis: phosphomonoester/-metabolites (PME, Peth, Pch); phospholipids breakdown: phosphodiester/-metabolites (PDE, Gpeth, Gpch); astrocyte activation: myo-Inositol (mI)) in 5 brain regions (dorsolateral prefrontal cortex, DLPFC; dorsomedial prefrontal cortex, DMPFC; dorsal anterior cingulate cortex, dACC; mediodorsal thalamus, Th; and hipoocampus, Hip). Psychopathology was assessed using the CAARMS-Interview as well as PANSS, BPRS-E and SCL-90-R ratings. Statistical analysis included multi-and univariate ANOVA, Kruskal-Wallis-tests and correlation analysis.

**Results:**

(i) In all UHR individuals (and also in the AS and BLIPS subgroup), NAA was reduced in the left Th. There was no alteration of Glu. While PCr was increased in the left DLPFC, left dACC (right trend) and in the right Hip, ATP was not different from controls. PME were decreased in the right Hip, PDE did not differ from controls. mI was found increased in the left Hip. (ii) In the GR subgroup PCr was increased in the bilateral Th. The PME metabolite Peth was decreased in the right Th. PDE were increased in the left dACC. mI was increased in the left Th.

**Discussion:**

While the observed pattern of metabolite abnormalities in the AS and BLIPS risk group suggests a pathology that affects the left thalamus (NAA decrease), left DLPFC, dACC and bilateral Hip (left: PCr increase, PME decrease; right mI increase), the pathology of the GR group appears more focussed on the bilateral Th (bil. PCr increase, right PME decrease, left mI increase) and left dACC (PDE increase). The results suggest a functional disturbance of networks including the left DLPFC, dACC, bilateral Hip and Th, whereby the latter might be more an expression of a genetic risk profile.

